# The Exosome Landscape in Acute Myeloid Leukemia: From Molecular Mechanisms to Translational Frontiers

**DOI:** 10.3390/genes17030290

**Published:** 2026-02-27

**Authors:** Elizabeth Vargas-Castellanos, Dayana Barbosa-Lopéz, Jair Figueroa-Emiliani

**Affiliations:** Hospital Universitario Mayor-Méderi, Universidad del Rosario, Bogota 111411, Colombia

**Keywords:** acute myeloid leukemia, exosomes, miRNAs, molecular, immune response

## Abstract

Acute myeloid leukemia (AML) is a biologically heterogeneous hematologic malignancy arising from the oncogenic transformation of hematopoietic stem and progenitor cells, resulting in clonal expansion and progressive subclonal diversification. Although considerable advances have deepened our understanding of AML pathogenesis, major challenges persist, particularly regarding relapses and therapeutic resistance. In recent years, exosomes—extracellular vesicles of 30–150 nm in diameter of endosomal origin—have emerged as critical mediators of intercellular communication within the AML tumor microenvironment. These vesicles transport a diverse cargo of proteins, metabolites, and nucleic acids, including mRNA, non-coding RNA species, and DNA, which is selectively packaged during their biogenesis. Circulating exosomes have garnered attention as promising liquid biomarkers for diagnosis, prognosis, and monitoring minimal residual disease, while also representing potential therapeutic targets or delivery platforms. Nonetheless, significant knowledge gaps remain regarding the mechanisms governing exosome biogenesis, cargo selection, and the functional impact on leukemia progression and immune modulation. This review focuses on the role of exosomes in acute myeloid leukemia, with an emphasis on the molecular mechanisms underlying their involvement in pathogenesis, tumor communication, and resistance to therapies, as well as their potential as diagnostic biomarkers.

## 1. Introduction

Acute myeloid leukemia (AML) is a bone marrow disorder that affects hematopoietic stem cells. AML is characterized by the abnormal clonal expansion of immature myeloblasts with impaired differentiation, accumulating in the bone marrow and peripheral blood and impairing normal hematopoiesis [1]. Acute leukemia is more common in adults and presents a significant clinical challenge due to its considerable biological diversity and tendency to relapse [2]. In Latin America, although the general prevalence of leukemia is moderate, countries such as Peru, Ecuador and Colombia have the highest rates in the region, with 8.1, 6.5 and 6.1 cases per 100,000 inhabitants, respectively [3].

In recent decades, advances in research have substantially expanded our understating of the pathogenesis of AML [4,5,6]. Although many questions remain, it is now recognized that AML develops from the oncogenic transformation of hematopoietic stem cells or from progenitor populations with a similar phenotype, which retain transcriptional programs characteristic of stem cells [7]. While the disease originates from a single malignant cell, the progressive accumulation of somatic mutations in its descendants, together with the selective pressure exerted by the bone marrow microenvironment, leads to marked genetic heterogeneity and fosters divergent subclonal evolution [8,9].

In this context, exosomes derived from the endosome have emerged as key mediators of intercellular communication in the tumor ecosystem of AML [10]. Exosomes may contain a wide variety of molecules, including membrane proteins, cytosolic and nuclear proteins, extracellular matrix components, metabolites, as well as nucleic acids such as mRNA, different species of non-coding RNA, and DNA [11,12]. However, even when analyzing a purified population of exosomes, not all contain the same amount or type of load [13]. In addition, a selective enrichment of certain proteins and nucleic acids in exosomes has been observed with respect to their origin cell, suggesting the existence of specific mechanisms of selection and molecular packaging during their biogenesis [14].

The identification of exosomes in plasma has opened a new perspective in the development of liquid biomarkers for the diagnosis, prognosis, and monitoring of minimal residual disease [15]. At the same time, research has explored therapeutic strategies that aim to block their biogenesis, modify their release, or utilize them as delivery vehicles for drugs and immunomodulatory agent molecules [16,17].

Despite these advances, significant challenges remain in the comprehensive understanding of exosome biology in AML. This review focuses on the role of exosomes in AML, with an emphasis on the molecular mechanisms underlying their involvement in pathogenesis, tumor communication and resistance to therapies, as well as its potential as diagnostic and therapeutic tools.

## 2. Biogenesis and Biology of Exosomes

### 2.1. Mechanisms of Formation: Endosomes, ESCRT Complex, Rab GTPases

Exosomes are extracellular vesicles (EVs) 30–150 nm in diameter, originating from the endosomal pathway, which act as important mediators in intercellular communication by transporting metabolites, proteins, lipids, and various types of nucleic acids, including DNA, RNA, microRNA (miRNA), and long non-coding RNA (lncRNA) [18]. Due to their ability to mediate the transfer of intercellular biological information, exosomes play a fundamental role in modulating the immune response, regulating physiological processes, and influencing the molecular mechanisms involved in cancer development and progression [19].

**Figure 1:**
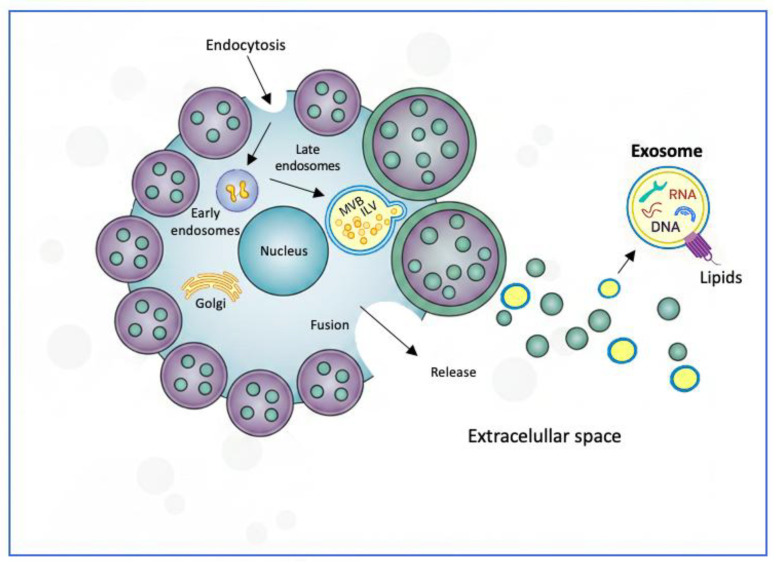
Exosome biogenesis.

Exosome biogenesis begins at the plasma membrane, where the cell incorporates components via endocytosis, forming early endosomes [20]. These progressively mature into late endosomes, within which the invagination of the endosomal membrane occurs to generate intraluminal vesicles (ILVs) [21]. This process is dependent on the ESCRT (Endosomal Sorting Complexes Required for Transport) machinery or alternative pathways mediated by specific lipid components, tetraspanins, and proteins [20]. Late endosomes containing ILVs are called multivesicular bodies (MVBs). When MVBs fuse with the cell membrane, they release ILVs into the extracellular space, at which point the vesicles are referred to as exosomes [22] (Figure 1).

### 2.2. Molecular Loading of Exosomes

The molecular cargo of exosomes results from a selective classification process that includes a wide diversity of biomolecules. These vesicles can carry membrane proteins, such as tetraspanins, integrins, and major histocompatibility complex (MHC) molecules, as well as cytosolic proteins, including kinases, transcription factors, and heat shock proteins [11,23]. In addition, exosomes contain bioactive lipids and nucleic acids, including different types of RNAs (miRNAs, lncRNAs, circRNAs, and mRNAs) and Single-stranded (ssDNA) and Double-stranded (dsDNA) fragments, along with low-molecular-weight metabolites. The selective incorporation of this load is regulated by mechanisms dependent and independent of the ESCRT complex, as well as by the action of tetraspanins and specialized lipid domains [14].

#### 2.2.1. Non-Coding RNA (miRNAs and lncRNAs)

miRNAs have been proposed as diagnostic, prognostic, and predictive biomarkers in various pathologies. Exosomes carry miRNAs that act as mediators of intercellular communication in the tumor microenvironment, modulating the gene expression of the receptor cells [24]. These miRNAs in the tumor microenvironment can promote oncogenic processes: (i) remodeling cell composition, (ii) improving angiogenesis, (iii) increasing proliferation and metastatic potential, (iv) transferring molecules related to drug resistance, (v) exerting tumor-suppressive functions [25].

LncRNAs are RNA molecules of more than 200 nucleotides that do not code for proteins but play essential regulatory functions in multiple cellular processes. LncRNAs present in exosomes are involved in proliferation, angiogenesis, metastasis, drug resistance and tissue remodeling by various mechanisms, such as miRNA sequestration (sponge effect), chromatin complex modulation, and interaction with signaling proteins [26].

#### 2.2.2. DNA, Proteins, Metabolites

Exosomal DNA mainly includes ssDNA and dsDNA fragments, as well as mitochondrial DNA, which reflects the genomic state of the origin cell and may contribute to the exchange of genetic information in the tumor microenvironment [27]. Exosomal DNA has been shown to play a key role in immune response and cancer progression [14].

Proteins present in exosomes include both membrane molecules, tetraspanins, integrins, and MHC complexes, as well as cytosolic proteins involved in cell signaling processes, stress response, metabolism, and cytoskeletal remodeling [19]. These proteins can influence tumor growth through multiple mechanisms: (i) They can act directly on cancer cells promoting proliferation, invasion and metastasis. (ii) They can modulate the tumor microenvironment, indirectly affecting tumor progression. (iii) Also, some proteins carried by exosomes contribute to regulating the immune response, inhibiting the activity of the immune system against the tumor and promoting immune evasion [19].

Low-molecular-weight metabolites transported in exosomes include lipids such as phospholipids, cholesterol, and triacylglycerols, as well as energy metabolism intermediates and regulatory molecules that can modify the metabolic activity of recipient cells [28]. Taking together, this diversity of biomolecules confirms that exosomes serve as information transfer platforms that significantly influence physiological and pathological processes, including cancer [19].

## 3. Exosomes in the Pathophysiology of AML

Recent research indicates that the bidirectional molecular interaction between leukemic stem cells (LSCs), leukemic blasts, and bone marrow niche stromal cells plays a key role in leukemogenesis, treatment resistance, and disease relapse [29,30,31]. In AML, exosomes derived from leukemic cells promote the survival and activity of LSCs and their progenitor cells through autocrine mechanisms [32], while inducing remodeling of the bone marrow niche through paracrine signals [33]. Several studies have described that these vesicles participate in key processes such as the activation of proliferative signals, the evasion of senescence and apoptosis, drug resistance, angiogenesis, immunosuppression, metabolic alterations, and the suppression of normal hematopoiesis [1,34].

Szczepanski et al. found that the plasma of patients with AML is enriched in exosomes compared with that of controls. Newly diagnosed AML patients exhibit significantly elevated plasma exosome levels [35]. The available evidence indicates that exosomes generated in the bone marrow microenvironment, as well as those migrating to this niche, are functionally primed to interact with stromal components, vasculature, and immune cells. These vesicles disrupt the normal functions of stromal, endothelial, and immune cells, transforming them into active support for the survival and expansion of leukemic cells. Consequently, exosomes promote a profound and sustained reorganization of the physiological bone marrow microenvironment, creating an environment conducive to cancer progression [36].

AML can reprogram the bone marrow microenvironment to favor leukemic cell expansion and suppress normal hematopoiesis, largely through the secretion of exosomes [33]. These extracellular vesicles released by AML cells increase dickkopf1 (Dkk1) expression in bone marrow mesenchymal stromal cells, thereby inhibiting physiological hematopoiesis by disrupting the WNT signaling pathway [37].

The WNT/β-catenin pathway plays a fundamental role in regulating the self-renewal, proliferation, and differentiation of hematopoietic stem cells. Under physiological conditions, the activation of WNT ligands stabilizes cytoplasmic β-catenin, allowing its translocation to the nucleus, where it regulates the transcription of genes involved in hematopoiesis [38]. However, DKK1 acts as an inhibitor of this pathway by blocking the interaction between WNT ligands and their receptors (LRP5/6), promoting β-catenin degradation and suppressing WNT signaling. Consequently, normal hematopoietic differentiation is impaired, creating an environment conducive to the expansion of leukemic cells [39].

Furthermore, AML-derived exosomes activate vascular endothelial growth factor (VEGF) signaling in human umbilical vein endothelial cells (HUVECs) by transferring angiogenic factors, proteins, and miRNAs, promoting the formation of vascular tubular structures and favoring tumor angiogenesis [40,41].

One of the key mechanisms by which exosomes contribute to the development of leukemia is through the transport of oncogenic RNAs, particularly miRNAs, to hematopoietic stem cells [26,42]. These miRNAs can reprogram gene expression in recipient cells, altering their phenotype, promoting abnormal self-renewal and resistance to apoptosis, and facilitating their transformation into leukemic cells. In this manner, exosomes serve as mediators of intercellular communication within the bone marrow microenvironment, thereby promoting disease progression and the clonal expansion of malignant cells [42].

### 3.1. Functional Impact of Exosomal miRNAs in AML

One of the main advantages of exosomes is their role as carriers of biologically active molecules. Exosome-derived miRNAs are protected from degradation by extracellular ribonucleases, making them stable, reliable, and reproducible biomarkers for disease detection and monitoring [43]. Studies have shown that exosomes, particularly their miRNAs, can be detected stably and in significant concentrations in body fluids, such as blood and urine [44]. In addition, specific protein and miRNA expression profiles have been identified in exosomes that are closely related to AML [45].

Several miRNAs enriched in AML exosomes have been implicated in disease progression. Fang et al. demonstrated that miR-10b is significantly upregulated in plasma-derived exosomes from patients with newly diagnosed AML. This miRNA contributes to leukemogenesis by inhibiting granulocytic and monocytic differentiation while promoting the proliferation of immature myeloid cells. Consequently, elevated serum levels of miR-10b are closely associated with poor prognosis [42].

Another exosomal miRNA with functional relevance in AML is miR-4532, which targets CD34^+^ hematopoietic stem cells and suppresses colony-forming capacity through the inhibition of Leucine Zipper Downregulated in Cancer 1 (LDOC1). This suppression leads to the enhanced activation of the JAK2/STAT3 signaling pathway, promoting the abnormal proliferation of leukemic cells. These results suggest miR-4532 and tumor-derived exosomal miRs as future alternative targets in the therapy of AML [46]. Similarly, miR-26a-5p, derived from mesenchymal stem cell exosomes in AML patients, enhances leukemic cell proliferation, migration, and invasion by targeting Glycogen Synthase Kinase 3 beta (GSK3β), thereby activating the WNT/β-catenin pathway [47].

Another elevated miRNA in patients with AML is exosomal miR-92a-3p. It has been implicated in regulating the chemotherapeutic response by inhibiting PTEN and activating β-catenin signaling, which could contribute to cytarabine resistance and disease progression [48]. These findings highlight miR-92a-3p as a candidate biomarker of resistance and poor clinical prognosis in AML, although further studies are needed to clarify its exact roles in leukemogenesis.

**Figure 2:**
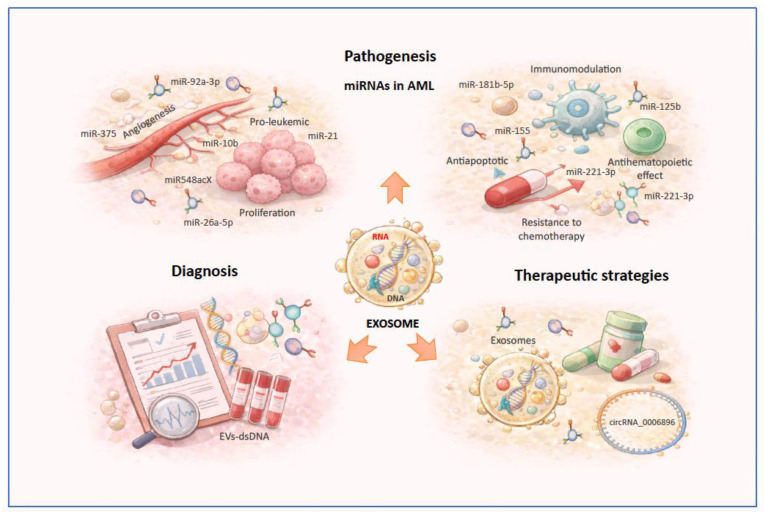
Overview of the functional roles of exosome-associated in AML. Leukemia-derived exosomes transport diverse RNA species, including miRNAs, circRNAs, and dsDNA, which contribute to disease pathogenesis by promoting proliferation, angiogenesis, immune evasion, and chemoresistance.

Collectively, these findings highlight that exosomal miRNAs are active mediators of AML progression. By remodeling the bone marrow niche, suppressing normal hematopoiesis, promoting angiogenesis, and facilitating immune evasion, exosomal miRNAs contribute to a microenvironment that favors the survival and expansion of leukemic cells (Table 1). Despite persistent gaps in our understanding of the biological and molecular mechanisms of exosomal miRNAs in AML, existing studies underscore the critical role of exosomes in AML pathogenesis (Figure 2).

### 3.2. Impact on Exosomal RNA in AML

Exosomes contain a diverse repertoire of nucleic acids, including mRNA, miRNAs, ribosomal RNA, and lncRNA [62,63]. Notably, exosome RNAs function as carriers of genetic information capable of modulating protein expression and biological responses in recipient cells. Accumulating evidence indicates that the RNA composition of exosomes differs from that of their parental cells, supporting the presence of selective RNA sorting mechanisms during exosome biogenesis [51].

Huan et al. investigated the role of exosomes in shaping the AML niche within the bone marrow microenvironment, with a particular focus on exosome biogenesis and RNA trafficking. They demonstrated that both primary AML cells and AML cell lines release exosome-sized vesicles that are efficiently taken up by recipient cells. These exosomes were enriched in multiple coding and non-coding RNAs relevant to AML pathogenesis. Notably, the uptake of AML-derived exosomes by bone marrow stromal cells altered their secretion of growth factors. Collectively, these findings revealed that AML exosome-mediated RNA trafficking reprograms the proliferative, angiogenic, and migratory responses of hematopoietic progenitor and stromal cells, providing insight into how the bone marrow niche is remodeled during AML infiltration [64].

Building on the evidence that AML-derived exosomes reprogram the bone marrow microenvironment through RNA transfer, subsequent studies further demonstrated the functional consequences of this exosome-mediated communication on hematopoietic regulation [33]. AML cell lines and patient-derived blasts were shown to release exosomes enriched in RNA and protein cargo that induce proangiogenic changes in recipient cells. Extending these observations in vitro assays and murine xenograft models revealed that AML-derived exosomes downregulate critical stromal retention factors, including stem cell factor (SCF) and CXCL12, leading to the mobilization of hematopoietic stem and progenitor cells from the bone marrow. Moreover, exosomes directly impaired hematopoietic stem and progenitor cell function, as evidenced by reduced clonogenicity, the loss of CXCR4 and c-Kit expression, and the repression of key hematopoietic transcription factors such as c-MYB, CEBP-β, and HOXA9 [65]. Notably, these effects occurred independently of direct leukemia–stromal cell contact, underscoring the central role of paracrine exosome trafficking in niche remodeling and the suppression of residual hematopoiesis during AML progression [64].

A study in single-cell RNA sequencing analysis revealed a significant upregulation of genes associated with cellular invasion, motility, and clonal expansion in AML patients compared with healthy donors, suggesting that exosome-mediated signaling may play a critical role in enhancing leukemic cell aggressiveness and driving disease progression [66]. Overall, these findings underline that exosome RNA is a key factor in AML progression, mediating paracrine reprogramming of the bone marrow microenvironment to promote leukemic expansion, angiogenesis, and the suppression of normal hematopoiesis. This exosome-mediated RNA traffic increases leukemic aggressiveness, regardless of direct cell–cell contact, highlighting exosomes RNA as a critical regulator in AML.

### 3.3. Impact of Exosomal dsDNA in AML

Considering the limitations associated with minimal residual disease detection in AML, there has been a sustained effort to identify reliable biomarkers that can accurately reflect disease persistence [67]. Within this framework, exosomes have gained increasing attention due to their emerging diagnostic and prognostic value in cancer, positioning them as promising surrogate indicators of disease presence and progression [68].

Kontopoulou et al. evaluated the diagnostic potential of EVs-dsDNA in pediatric AML using plasma samples from both bone marrow and peripheral blood. In their study, EVs were isolated from plasma of 29 pediatric AML patients at initial diagnosis or during treatment, followed by dsDNA extraction and analysis of leukemia-specific mutations using next-generation sequencing (NGS). EVs-dsDNA accurately mirrored leukemia-specific mutations identified in genomic DNA from primary leukemic cells in 18 of 20 patients. Moreover, nanoparticle tracking analysis revealed a significant reduction in EV numbers after treatment compared with diagnosis, and leukemia-associated mutations became undetectable in EVs-dsDNA in most post-treatment samples [69]. These findings support the use of leukemia-derived EVs-dsDNA as a minimally invasive tool for assessing mutational status and monitoring treatment response in AML (Figure 2).

Bernardi et al. conducted a pilot study evaluating the feasibility of NGS analysis of dsDNA derived from leukemia-enriched exosomes in adult patients with AML. Exosomes were isolated from plasma samples of 14 adult AML patients, and leukemia-derived exosome fractions were specifically analyzed. The number and type of detected mutations were compared with those identified in bone marrow and peripheral blood cells. The mutational profile of exosomes dsDNA demonstrated a high degree of concordance with cellular DNA, with 86.5% homology to bone marrow-derived DNA and 75% to peripheral blood-derived DNA [70]. Collectively, these findings support the feasibility of leukemia-derived exosome enrichment followed by exosomal dsDNA NGS analysis as a minimally invasive liquid biopsy approach for AML biomarker detection and for identifying active leukemic cells residing in the bone marrow.

Altogether, growing evidence supports exosomal dsDNA as a robust and biologically relevant biomarker in AML. Exosomal dsDNA mirrors the mutational landscape of leukemic cells and demonstrates high concordance with bone marrow and peripheral blood genomic DNA, while offering the advantage of minimally invasive sampling. Its association with leukemic burden and its dynamic changes during treatment underscore its potential utility for mutational profiling, disease monitoring, and assessment of therapeutic response [71]. Collectively, these findings position exosomal dsDNA as a promising liquid biopsy tool that may contribute to the more precise and personalized management of AML patients.

### 3.4. AML-Derived Exosomes and Modulation of the Immune Response

Communication between leukemic cells and immune system cells is crucial for understanding disease progression. In this context, exosomes derived from AML cells have been shown to exert a significant immunosuppressive effect, contributing to the evasion of the immune system by tumor cells [51].

Circulating exosomes can transfer immunosuppressive payloads to recipient immune cells, inhibiting their antitumor activities. Chan et al. demonstrated that exosomes isolated from the pretreatment plasma of AML patients undergoing adoptive cell therapy with NK-92 cells block the antileukemic cytotoxicity and other effector functions of these cells. Unlike other cell communication mechanisms, NK-92 cells do not internalize AML-derived exosomes; instead, these exosomes interact with surface receptors expressed on NK-92 cells, simultaneously providing multiple inhibitory ligands. These signals are processed intracellularly and lead to the activation of various suppressor pathways, resulting in the functional reprogramming of NK-92 cells. Consequently, their cytotoxic capacity and therapeutic potential are reduced [72].

Furthermore, AML exosomes carry multiple immunoinhibitory molecules capable of directly suppressing natural killer (NK) cell activation and inhibiting their cytotoxic activity [73]. In particular, AML cell-derived exosomes have been shown to exert their immunosuppressive effects by activating the PD-1/PD-L1 signaling pathway [74]. The interaction between PD-L1 present in tumor exosomes and the PD-1 receptor expressed on NK cells leads to the inhibition of immune activation signals, thereby reducing these cells’ ability to recognize and eliminate leukemic cells. As a result, AML exosomes promote a state of immunotolerance that favors tumor survival and disease progression [75].

NK cells play a fundamental role in immune surveillance against cancer; however, their function is altered in many hematologic malignancies. The Natural Killer Group 2D (NKG2D) activating receptor, expressed on NK cells, NK T cells, and CD8^+^ T lymphocytes, is frequently inhibited in the tumor context, significantly contributing to immune evasion mechanisms [76]. In patients with AML, exosomally transported TGF-β1 suppresses NKG2D expression, markedly reducing NK cell-mediated cytotoxicity [35,77]. Consequently, circulating exosomes in the plasma of AML patients exert a modulatory effect on innate immunity. In this context, IL-15 can counteract this suppression by interfering with the SMAD signaling pathway and preserving NK cell function [77]. Additionally, AML-derived exosomes promote a state of immunological tolerance in dendritic cells (DCs), a key process for tumor evasion [78]. These tumor-derived exosomes inhibit both DC differentiation and functional activity, largely due to the immunosuppressive action of TGF-β [79].

Finally, exosomes derived from leukemic cells in AML can modulate the differentiation and polarization of immune cells, promoting the development of immunosuppressive phenotypes that favor tumor progression [80]. These exosomes are capable of reprogramming T cells and myeloid cells into functional states that reduce the effective antitumor response, contributing to an immunosuppressive microenvironment [81]. Furthermore, adverse conditions in the tumor microenvironment, such as hypoxia, can increase the release of exosomes enriched in immunomodulatory miRNAs, which induce the polarization of monocytes toward M2 macrophages with protumor functions [82,83]. Taken together, these mechanisms facilitate immune evasion by leukemic cells and limit the efficacy of antileukemic immune responses.

### 3.5. Exosomes: Hidden Allies of Therapeutic Resistance

One of the functions of exosomes that has not yet been fully characterized is their ability to promote the survival of leukemic cells during chemotherapy. In this context, Viola et al. evaluated the chemoresistance effect of exosomes derived from leukemic stem cells in AML, identifying that miR-155 and miR-375 transported by leukemic exosomes induce a state of chemoresistance in AML cells. Furthermore, they demonstrated that AML-derived exosomes exert their chemoprotective effect by modulating TGF-β levels [60]. TGF-β1 is a multifunctional cytokine that plays an important role in tumor biology. During the early stages of carcinogenesis, it usually exercises suppressive functions by restricting cell proliferation and promoting apoptosis [84]. In the context of AML, circulating plasma-derived exosomes of patients have been shown to reduce the cytotoxicity of natural killer cells through the negative regulation of the NKG2D activator receptor, thus compromising antitumor immunological surveillance [35]. Further studies by Tohumeken et al. reported that AML-derived exosomes induce the reprogramming of monocytes to myeloid-derived suppressor cells through the activation of the TLR2/Akt/mTOR signaling pathway, reinforcing an immunosuppressive microenvironment favorable to disease progression [85].

Exosomes released by AML cells also promote angiogenesis by stimulating the proliferation, migration, and tube-forming activity of human umbilical vein endothelial cells [51]. In turn, these activated endothelial cells confer chemoresistance to leukemic cells, reinforcing a protective tumor niche. These exosomes contain VEGF mRNA and its receptor VEGFR, induce VEGFR expression, and increase glycolysis in endothelial cells, promoting their survival and resistance to apoptosis [86]. Collectively, AML exosomes contribute to vascular remodeling and chemotherapy resistance, representing a potential therapeutic target.

Recent studies have expanded our understanding of leukemic cell-derived exosomes in the progression of AML and in therapeutic resistance. One recent study demonstrated that the circular RNA (circRNA) circ_0006896 is highly expressed in leukemic cells and exosomes from AML patients, and is associated with a worse clinical prognosis. This circRNA promotes cell proliferation, reduces sensitivity to chemotherapy, and interferes with the cytotoxic function of CD8 T lymphocytes [87]. CircRNAs are characterized by a covalently closed loop structure, resulting in RNA molecules that are more stable than linear RNAs [88]. These studies reinforce the central role of exosomes as mediators of therapeutic resistance, immune evasion, and tumor progression in AML, and position them as promising targets for the development of new therapeutic strategies (Figure 2).

Beyond their value as biomarkers, exosomes represent a clinically relevant therapeutic platform in AML. Multiple studies have demonstrated that AML-derived exosomes actively shape disease biology by remodeling the bone marrow microenvironment, suppressing anti-leukemic immune responses, and impairing normal hematopoiesis through the transfer of oncogenic miRNAs and proteins [45,89]. Importantly, these same biological properties can be exploited therapeutically. Engineered exosomes could function as “Trojan horse” delivery systems to selectively target leukemic cells and deliver therapeutic cargo, including tumor-suppressive miRNAs, siRNAs, or small-molecule drugs, with high specificity and reduced systemic toxicity [90,91,92]. Such exosome-based strategies offer a promising translational avenue to modulate leukemogenic pathways, overcome drug resistance, and enhance treatment precision in AML, supporting their potential incorporation into future therapeutic approaches.

## 4. Conclusions

In conclusion, exosomes derived from leukemic cells play multiple roles in AML, acting not only as potential diagnostic biomarkers but also as key mediators of intercellular communication within the bone marrow niche. These extracellular vesicles contribute significantly to the pathogenesis of the disease by inducing immunosuppression and promoting the invasion and dissemination of leukemic cells.

Exosomal interference with immune cells used in therapeutic strategies represents an additional mechanism of tumor resistance and highlights the need to develop approaches aimed at neutralizing the immunosuppressive effects of exosomes to improve clinical outcomes in AML. Furthermore, blocking the PD-1/PD-L1 pathway emerges as a promising strategy to reverse exosome-induced immunosuppression, restore the cytotoxic function of NK cells, and enhance the efficacy of immunotherapies.

Taken together, these findings highlight the central role of exosomes in AML biology and underscore their potential as diagnostic, prognostic, and therapeutic tools. However, further studies are needed to better understand the biological and molecular mechanisms that regulate their functions in AML.

## Figures and Tables

**Table 1:** Functional impact of exosome-derived miRNAs in AML.

Exosomal miRNA	Cell of Origin	Main Function	Effect	Ref.
miR-21	Leukemic cells	Antiapoptotic	Increases leukemic survival and resistance to chemotherapy	[49,50]
miR-10b	Leukemic cells	Pro-leukemic	Inhibit the differentiation of hematopoietic stem cells while enhancing the proliferative capacity of immature myeloid cells	[42,51]
miR-155	Leukemic cells	Post-transcriptional regulation	Regulation of key genes, especially those involved in hematopoietic differentiation and survival	[10,52,53]
miR-125b	Leukemic cells	Antiapoptotic	Suppresses pro-apoptotic genes, favoring the survival and proliferation of leukemic blasts	[54,55]
miR-181b-5p	Leukemic cells	Antiapoptotic	Capable of modulating cell cycle and suppressing apoptosis	[55,56]
miR-221-3p	Leukemic cells	Antiapoptotic	Capable of modulating cell cycle and suppressing apoptosis	[57,58]
miR548ac	Leukemic cells	Antihematopoietic effect	Suppression of hematopoietic stem cells	[51,59]
miR-26a-5p	Leukemic cells	Pro-leukemic	Activates the WNT/β-catenin pathway in AML cells, promoting tumor progression	[47]
miR-375	Leukemic cells	Antiapoptotic	Induction of chemoresistance through altering the level of TGF-β	[60,61]
miR-92a-3p	Leukemic cells	Pro-leukemic	Regulation of chemotherapy resistance and oncogenic signaling (WNT/β-catenin)	[48]

## Data Availability

No new data were created or analyzed in this study. Data sharing is not applicable to this article.

## Abbreviations

AML	Acute myeloid leukemia
miRNA	microRNA
lncRNA	long non-coding RNA
ILVs	Intraluminal vesicles
ESCRT	Endosomal Sorting Complexes Required for Transport
MVBs	Multivesicular bodies
MHC	Major histocompatibility complex
LSCs	Leukemic stem cells
Dkk1	Dickkopf1
VEGF	Vascular endothelial growth factor
HUVECs	Human umbilical endothelial cells
LDOC1	Leucine Zipper Downregulated in Cancer 1
GSK3β	Glycogen Synthase Kinase 3 beta
TGF-β	Transforming Growth Factor beta
NK	Natural killer
NKG2D	Natural Killer Group 2D
DCs	Dendritic cells
ssDNA	Single-stranded
dsDNA	Double-stranded
circRNA	circular RNA
NGS	Next-generation sequencing
SCF	Stem cell factor

## References

[1] Li Q., Wang M., Liu L. (2023). The role of exosomes in the stemness maintenance and progression of acute myeloid leukemia. Biochem. Pharmacol..

[2] Grimwade D., Ivey A., Huntly B.J.P. (2016). Molecular landscape of acute myeloid leukemia in younger adults and its clinical relevance. Blood.

[3] Bray F., Laversanne M., Sung H., Ferlay J., Siegel R.L., Soerjomataram I., Jemal A. (2024). Global cancer statistics 2022: GLOBOCAN estimates of incidence and mortality worldwide for 36 cancers in 185 countries. CA Cancer J. Clin..

[4] Newell L.F., Cook R.J. (2021). Advances in acute myeloid leukemia. BMJ.

[5] Bullinger L., Döhner K., Döhner H. (2017). Genomics of Acute Myeloid Leukemia Diagnosis and Pathways. J. Clin. Oncol..

[6] Hou H.-A., Tien H.-F. (2020). Genomic landscape in acute myeloid leukemia and its implications in risk classification and targeted therapies. J. Biomed. Sci..

[7] Shlush L.I., Mitchell A., Heisler L., Abelson S., Ng S.W.K., Trotman-Grant A., Medeiros J.J.F., Rao-Bhatia A., Jaciw-Zurakowsky I., Marke R. (2017). Tracing the origins of relapse in acute myeloid leukaemia to stem cells. Nature.

[8] Chopra M., Bohlander S.K. (2019). The cell of origin and the leukemia stem cell in acute myeloid leukemia. Genes Chromosomes Cancer.

[9] Lapidot T., Sirard C., Vormoor J., Murdoch B., Hoang T., Caceres-Cortes J., Minden M., Paterson B., Caligiuri M.A., Dick J.E. (1994). A cell initiating human acute myeloid leukaemia after transplantation into SCID mice. Nature.

[10] Hornick N.I., Doron B., Abdelhamed S., Huan J., Harrington C.A., Shen R., Cambronne X.A., Verghese S.C., Kurre P. (2016). AML suppresses hematopoiesis by releasing exosomes that contain microRNAs targeting c-MYB. Sci. Signal..

[11] Keerthikumar S., Chisanga D., Ariyaratne D., Al Saffar H., Anand S., Zhao K., Samuel M., Pathan M., Jois M., Chilamkurti N. (2016). ExoCarta: A Web-Based Compendium of Exosomal Cargo. J. Mol. Biol..

[12] Pathan M., Fonseka P., Chitti S.V., Kang T., Sanwlani R., Van Deun J., Hendrix A., Mathivanan S. (2019). Vesiclepedia 2019: A compendium of RNA, proteins, lipids and metabolites in extracellular vesicles. Nucleic Acids Res..

[13] Chevillet J.R., Kang Q., Ruf I.K., Briggs H.A., Vojtech L.N., Hughes S.M., Cheng H.H., Arroyo J.D., Meredith E.K., Gallichotte E.N. (2014). Quantitative and stoichiometric analysis of the microRNA content of exosomes. Proc. Natl. Acad. Sci. USA.

[14] Kalluri R., LeBleu V.S. (2020). The biology, function, and biomedical applications of exosomes. Science.

[15] Hong C.S., Muller L., Whiteside T.L., Boyiadzis M. (2014). Plasma exosomes as markers of therapeutic response in patients with acute myeloid leukemia. Front. Immunol..

[16] Lee J.-H.H.J., Lee J.-H.H.J., Chakraborty K., Hwang J., Lee Y.-K.K. (2022). Exosome-based drug delivery systems and their therapeutic applications. RSC Adv..

[17] Gutierrez-Millan C., Calvo Díaz C., Lanao J.M., Colino C.I. (2021). Advances in Exosomes-Based Drug Delivery Systems. Macromol. Biosci..

[18] Théry C. (2011). Exosomes: Secreted vesicles and intercellular communications. F1000 Biol. Rep..

[19] Chen Y.-F.F., Luh F., Ho Y.-S.S., Yen Y. (2024). Exosomes: A review of biologic function, diagnostic and targeted therapy applications, and clinical trials. J. Biomed. Sci..

[20] Kalluri R. (2016). The biology and function of exosomes in cancer. J. Clin. Investig..

[21] Ma Y., Brocchini S., Williams G.R. (2023). Extracellular vesicle-embedded materials. J. Control. Release.

[22] Xu M., Ji J., Jin D., Wu Y., Wu T., Lin R., Zhu S., Jiang F., Ji Y., Bao B. (2023). The biogenesis and secretion of exosomes and multivesicular bodies (MVBs): Intercellular shuttles and implications in human diseases. Genes Dis..

[23] Lasda E., Parker R. (2016). Circular RNAs Co-Precipitate with Extracellular Vesicles: A Possible Mechanism for circRNA Clearance. PLoS ONE.

[24] Sun Z., Shi K., Yang S., Liu J., Zhou Q., Wang G., Song J., Li Z., Zhang Z., Yuan W. (2018). Effect of exosomal miRNA on cancer biology and clinical applications. Mol. Cancer.

[25] Wang S., Jin S., Zhang J., Wang X. (2025). Exosomal miRNAs: Key Regulators of the Tumor Microenvironment and Cancer Stem Cells. Int. J. Mol. Sci..

[26] Han Q.-F., Li W.-J., Hu K.-S., Gao J., Zhai W.-L., Yang J.-H., Zhang S.-J. (2022). Exosome biogenesis: Machinery, regulation, and therapeutic implications in cancer. Mol. Cancer.

[27] Ghanam J., Chetty V.K., Barthel L., Reinhardt D., Hoyer P.-F., Thakur B.K. (2022). DNA in extracellular vesicles: From evolution to its current application in health and disease. Cell Biosci..

[28] Nicolini A., Ferrari P., Biava P.M. (2021). Exosomes and Cell Communication: From Tumour-Derived Exosomes and Their Role in Tumour Progression to the Use of Exosomal Cargo for Cancer Treatment. Cancers.

[29] Hira V.V.V., Van Noorden C.J.F., Carraway H.E., Maciejewski J.P., Molenaar R.J. (2017). Novel therapeutic strategies to target leukemic cells that hijack compartmentalized continuous hematopoietic stem cell niches. Biochim. Biophys. Acta Rev. Cancer.

[30] Lane S.W., Wang Y.J., Lo Celso C., Ragu C., Bullinger L., Sykes S.M., Ferraro F., Shterental S., Lin C.P., Gilliland D.G. (2011). Differential niche and Wnt requirements during acute myeloid leukemia progression. Blood.

[31] Binder S., Luciano M., Horejs-Hoeck J. (2018). The cytokine network in acute myeloid leukemia (AML): A focus on pro- and anti-inflammatory mediators. Cytokine Growth Factor Rev..

[32] Record M., Carayon K., Poirot M., Silvente-Poirot S. (2014). Exosomes as new vesicular lipid transporters involved in cell-cell communication and various pathophysiologies. Biochim. Biophys. Acta.

[33] Kumar B., Garcia M., Weng L., Jung X., Murakami J.L., Hu X., McDonald T., Lin A., Kumar A.R., DiGiusto D.L. (2018). Acute myeloid leukemia transforms the bone marrow niche into a leukemia-permissive microenvironment through exosome secretion. Leukemia.

[34] Marchand T., Pinho S. (2021). Leukemic Stem Cells: From Leukemic Niche Biology to Treatment Opportunities. Front. Immunol..

[35] Szczepanski M.J., Szajnik M., Welsh A., Whiteside T.L., Boyiadzis M. (2011). Blast-derived microvesicles in sera from patients with acute myeloid leukemia suppress natural killer cell function via membrane-associated transforming growth factor-beta1. Haematologica.

[36] Boyiadzis M., Whiteside T.L. (2017). The emerging roles of tumor-derived exosomes in hematological malignancies. Leukemia.

[37] Wang W., Wu X., Zheng J., Yin R., Li Y., Wu X., Xu L., Jin Z. (2023). Utilizing exosomes as sparking clinical biomarkers and therapeutic response in acute myeloid leukemia. Front. Immunol..

[38] Elyamany G., Rizwan H., Akhter A., Aljabry M.S., Alotaibi S., Albalawi M.A.H., Shabani-Rad M.-T., Roshan T.M., Mansoor A. (2023). “Losing the Brakes”-Suppressed Inhibitors Triggering Uncontrolled Wnt/ß-Catenin Signaling May Provide a Potential Therapeutic Target in Elderly Acute Myeloid Leukemia. Curr. Issues Mol. Biol..

[39] Hu Y., Li S. (2016). Survival regulation of leukemia stem cells. Cell. Mol. Life Sci..

[40] Miyamoto K.N., Bonatto D. (2020). Circulating cells and exosomes in acute myelogenous leukemia and their role in disease progression and survival. Clin. Immunol..

[41] Mirfakhraie R., Noorazar L., Mohammadian M., Hajifathali A., Gholizadeh M., Salimi M., Sankanian G., Roshandel E., Mehdizadeh M. (2022). Treatment Failure in Acute Myeloid Leukemia: Focus on the Role of Extracellular Vesicles. Leuk. Res..

[42] Fang Z., Wang X., Wu J., Xiao R., Liu J. (2020). High serum extracellular vesicle miR-10b expression predicts poor prognosis in patients with acute myeloid leukemia. Cancer Biomark..

[43] Deng W., Wang L., Pan M., Zheng J. (2020). The regulatory role of exosomes in leukemia and their clinical significance. J. Int. Med. Res..

[44] Ha D., Yang N., Nadithe V. (2016). Exosomes as therapeutic drug carriers and delivery vehicles across biological membranes: Current perspectives and future challenges. Acta Pharm. Sin. B.

[45] Hornick N.I., Huan J., Doron B., Goloviznina N.A., Lapidus J., Chang B.H., Kurre P. (2015). Serum Exosome MicroRNA as a Minimally-Invasive Early Biomarker of AML. Sci. Rep..

[46] Zhao C., Du F., Zhao Y., Wang S., Qi L. (2019). Acute myeloid leukemia cells secrete microRNA-4532-containing exosomes to mediate normal hematopoiesis in hematopoietic stem cells by activating the LDOC1-dependent STAT3 signaling pathway. Stem Cell Res. Ther..

[47] Ji D., He Y., Lu W., Rong Y., Li F., Huang X., Huang R., Jiang Y., Chen G. (2021). Small-sized extracellular vesicles (EVs) derived from acute myeloid leukemia bone marrow mesenchymal stem cells transfer miR-26a-5p to promote acute myeloid leukemia cell proliferation, migration, and invasion. Hum. Cell.

[48] Li H., Xie C., Lu Y., Chang K., Guan F., Li X. (2022). Exosomal miR92a Promotes Cytarabine Resistance in Myelodysplastic Syndromes by Activating Wnt/β-catenin Signal Pathway. Biomolecules.

[49] Agha D.M., Rouas R., Najar M., Bouhtit F., Fayyad-Kazan H., Lagneaux L., Bron D., Meuleman N., Lewalle P., Merimi M. (2020). Impact of Bone Marrow miR-21 Expression on Acute Myeloid Leukemia T Lymphocyte Fragility and Dysfunction. Cells.

[50] Cariello M., Squilla A., Piacente M., Venutolo G., Fasano A. (2023). Drug Resistance: The Role of Exosomal miRNA in the Microenvironment of Hematopoietic Tumors. Molecules.

[51] Amin A.H., Al Sharifi L.M., Kakhharov A.J., Opulencia M.J.C., Alsaikhan F., Bokov D.O., Majdi H.S., Jawad M.A., Hammid A.T., Shalaby M.N. (2022). Role of Acute Myeloid Leukemia (AML)-Derived exosomes in tumor progression and survival. Biomed. Pharmacother..

[52] Izadirad M., Huang Z., Jafari F., Hamidieh A.A., Gharehbaghian A., Li Y.-D., Jafari L., Chen Z.-S. (2021). Extracellular Vesicles in Acute Leukemia: A Mesmerizing Journey With a Focus on Transferred microRNAs. Front. Cell Dev. Biol..

[53] Caivano A., La Rocca F., Simeon V., Girasole M., Dinarelli S., Laurenzana I., De Stradis A., De Luca L., Trino S., Traficante A. (2017). MicroRNA-155 in serum-derived extracellular vesicles as a potential biomarker for hematologic malignancies—A short report. Cell. Oncol..

[54] Jiang L., Deng T., Wang D., Xiao Y. (2018). Elevated Serum Exosomal miR-125b Level as a Potential Marker for Poor Prognosis in Intermediate-Risk Acute Myeloid Leukemia. Acta Haematol..

[55] Fazio M., Stagno F., Penna G., Mirabile G., Allegra A. (2025). Navigating the Landscape of Exosomal microRNAs: Charting Their Pivotal Role as Biomarkers in Hematological Malignancies. Non-Coding RNA.

[56] Kang K.-W., Gim J.-A., Hong S., Kim H.K., Choi Y., Park J.-H., Park Y. (2024). Use of extracellular vesicle microRNA profiles in patients with acute myeloid leukemia for the identification of novel biomarkers. PLoS ONE.

[57] Li M., Sun G., Zhao J., Pu S., Lv Y., Wang Y., Li Y., Zhao X., Wang Y., Yang S. (2024). Small extracellular vesicles derived from acute myeloid leukemia cells promote leukemogenesis by transferring miR-221-3p. Haematologica.

[58] Chow J.T.-S., Salmena L. (2024). “One way, or another, I’m gonna find ya”: miR-221-3p finds its targets via small extracellular vesicles. Haematologica.

[59] Zhao C., Zhao Y., Zhao J., Meng G., Huang S., Liu Y., Wang S., Qi L. (2022). Acute myeloid leukemia cell-derived extracellular vesicles carrying microRNA-548ac regulate hematopoietic function via the TRIM28/STAT3 pathway. Cancer Gene Ther..

[60] Viola S., Traer E., Huan J., Hornick N.I., Tyner J.W., Agarwal A., Loriaux M., Johnstone B., Kurre P. (2016). Alterations in acute myeloid leukaemia bone marrow stromal cell exosome content coincide with gains in tyrosine kinase inhibitor resistance. Br. J. Haematol..

[61] Agha D.M., Rouas R., Najar M., Bouhtit F., Naamane N., Fayyad-Kazan H., Bron D., Meuleman N., Lewalle P., Merimi M. (2020). Identification of Acute Myeloid Leukemia Bone Marrow Circulating MicroRNAs. Int. J. Mol. Sci..

[62] Dai J., Su Y., Zhong S., Cong L., Liu B., Yang J., Tao Y., He Z., Chen C., Jiang Y. (2020). Exosomes: Key players in cancer and potential therapeutic strategy. Signal Transduct. Target. Ther..

[63] Zhu L., Sun H.-T., Wang S., Huang S.-L., Zheng Y., Wang C.-Q., Hu B.-Y., Qin W., Zou T.-T., Fu Y. (2020). Isolation and characterization of exosomes for cancer research. J. Hematol. Oncol..

[64] Huan J., Hornick N.I., Shurtleff M.J., Skinner A.M., Goloviznina N.A., Roberts C.T., Kurre P. (2013). RNA trafficking by acute myelogenous leukemia exosomes. Cancer Res..

[65] Huan J., Hornick N.I., Goloviznina N.A., Kamimae-Lanning A.N., David L.L., Wilmarth P.A., Mori T., Chevillet J.R., Narla A., Roberts C.T. (2015). Coordinate regulation of residual bone marrow function by paracrine trafficking of AML exosomes. Leukemia.

[66] Jia J. (2025). TGF-β-Enriched Exosomes from Acute Myeloid Leukemia Activate Smad2/3–MMP2 and ERK1/2 Signaling to Promote Leukemic Cell Proliferation, Migration, and Immune Modulation. Curr. Issues Mol. Biol..

[67] Keller S., Ridinger J., Rupp A.-K., Janssen J.W.G., Altevogt P. (2011). Body fluid derived exosomes as a novel template for clinical diagnostics. J. Transl. Med..

[68] Boyiadzis M., Whiteside T.L. (2016). Plasma-derived exosomes in acute myeloid leukemia for detection of minimal residual disease: Are we ready?. Expert Rev. Mol. Diagn..

[69] Kontopoulou E., Strachan S., Reinhardt K., Kunz F., Walter C., Walkenfort B., Jastrow H., Hasenberg M., Giebel B., von Neuhoff N. (2020). Evaluation of dsDNA from extracellular vesicles (EVs) in pediatric AML diagnostics. Ann. Hematol..

[70] Bernardi S., Farina M., Bosio K., Di Lucanardo A., Leoni A., Re F., Polverelli N., Turra A., Morello E., Buttini E.A. (2022). Feasibility of Leukemia-Derived Exosome Enrichment and Co-isolated dsDNA Sequencing in Acute Myeloid Leukemia Patients: A Proof of Concept for New Leukemia Biomarkers Detection. Cancers.

[71] Bernardi S., Zanaglio C., Farina M., Polverelli N., Malagola M., Russo D. (2021). dsDNA from extracellular vesicles (EVs) in adult AML. Ann. Hematol..

[72] Hong C.-S., Sharma P., Yerneni S.S., Simms P., Jackson E.K., Whiteside T.L., Boyiadzis M. (2017). Circulating exosomes carrying an immunosuppressive cargo interfere with cellular immunotherapy in acute myeloid leukemia. Sci. Rep..

[73] Xie H., Wu Y., Huang J., Shen Q., Li X., Wang L., Lin J., Chi Z., Ke K., Lin X. (2025). NK Cell Exosomes Alleviate PD-L1 Expression and Facilitate Tumor Immunity by Repressing PI3K-AKT-mTOR Signaling. Immunol. Investig..

[74] Zhang L., Gajewski T.F., Kline J. (2009). PD-1/PD-L1 interactions inhibit antitumor immune responses in a murine acute myeloid leukemia model. Blood.

[75] Wang D., Zhou F., He L., Wang X., Song L., Wang H., Sun S., Guo Z., Ma K., Xu J. (2024). AML cell-derived exosomes suppress the activation and cytotoxicity of NK cells in AML via PD-1/PD-L1 pathway. Cell Biol. Int..

[76] Reiners K.S., Topolar D., Henke A., Simhadri V.R., Kessler J., Sauer M., Bessler M., Hansen H.P., Tawadros S., Herling M. (2013). Soluble ligands for NK cell receptors promote evasion of chronic lymphocytic leukemia cells from NK cell anti-tumor activity. Blood.

[77] Hong C.S., Muller L., Boyiadzis M., Whiteside T.L. (2014). Isolation and characterization of CD34+ blast-derived exosomes in acute myeloid leukemia. PLoS ONE.

[78] Benites B.D., Duarte A.d.S.S., Longhini A.L.F., Santos I., Alvarez M.C., Ribeiro L.N.d.M., de Paula E., Saad S.T.O. (2019). Exosomes in the serum of Acute Myeloid Leukemia patients induce dendritic cell tolerance: Implications for immunotherapy. Vaccine.

[79] Hedlund M., Nagaeva O., Kargl D., Baranov V., Mincheva-Nilsson L. (2011). Thermal- and oxidative stress causes enhanced release of NKG2D ligand-bearing immunosuppressive exosomes in leukemia/lymphoma T and B cells. PLoS ONE.

[80] Liu Y., Gu Y., Cao X. (2015). The exosomes in tumor immunity. Oncoimmunology.

[81] Raimondo S., Saieva L., Corrado C., Fontana S., Flugy A., Rizzo A., De Leo G., Alessandro R. (2015). Chronic myeloid leukemia-derived exosomes promote tumor growth through an autocrine mechanism. Cell Commun. Signal..

[82] Lee J.Y., Ryu D., Lim S.W., Ryu K.J., Choi M.E., Yoon S.E., Kim K., Park C., Kim S.J. (2021). Exosomal miR-1305 in the oncogenic activity of hypoxic multiple myeloma cells: A biomarker for predicting prognosis. J. Cancer.

[83] Mohseni A., Salehi F., Rostami S., Hadiloo K., Hashemi M., Baridjavadi Z., Ahangari F., Karami N., Samani F., Tahmasebi S. (2025). Harnessing the power of exosomes for diagnosis, prognosis, and treatment of hematological malignancies. Stem Cell Res. Ther..

[84] Zhang Y., Alexander P.B., Wang X.-F. (2016). TGF-β Family Signaling in the Control of Cell Proliferation and Survival. Cold Spring Harb. Perspect. Biol..

[85] Tohumeken S., Baur R., Böttcher M., Stoll A., Loschinski R., Panagiotidis K., Braun M., Saul D., Völkl S., Baur A.S. (2020). Palmitoylated Proteins on AML-Derived Extracellular Vesicles Promote Myeloid-Derived Suppressor Cell Differentiation via TLR2/Akt/mTOR Signaling. Cancer Res..

[86] Wang B., Wang X., Hou D., Huang Q., Zhan W., Chen C., Liu J., You R., Xie J., Chen P. (2019). Exosomes derived from acute myeloid leukemia cells promote chemoresistance by enhancing glycolysis-mediated vascular remodeling. J. Cell. Physiol..

[87] Can C., Yang X., Jia H., Wu H., Guo X., Wei Y., Jia Z., Liu W., Zhang A., He N. (2025). Exosomal circ_0006896 promotes AML progression via interaction with HDAC1 and restriction of antitumor immunity. Mol. Cancer.

[88] Verduci L., Strano S., Yarden Y., Blandino G. (2019). The circRNA-microRNA code: Emerging implications for cancer diagnosis and treatment. Mol. Oncol..

[89] Szewczyk G., Pyzlak M., Pankiewicz K., Szczerba E., Stangret A., Szukiewicz D., Skoda M., Bierła J., Cukrowska B., Fijałkowska A. (2021). The potential association between a new angiogenic marker fractalkine and a placental vascularization in preeclampsia. Arch. Gynecol. Obstet..

[90] ELAndaloussi S., Lakhal S., Mäger I., Wood M.J.A. (2013). Exosomes for targeted siRNA delivery across biological barriers. Adv. Drug Deliv. Rev..

[91] Herrmann I.K., Wood M.J.A., Fuhrmann G. (2021). Extracellular vesicles as a next-generation drug delivery platform. Nat. Nanotechnol..

[92] Mou D.-L., Jia Z.-S., Bai X.-F. (2005). Exosome: Trojan horse in immunotherapy. Sheng Li Ke Xue Jin Zhan.

